# Variability of Bacterial Essential Genes Among Closely Related Bacteria: The Case of *Escherichia coli*

**DOI:** 10.3389/fmicb.2018.01059

**Published:** 2018-05-29

**Authors:** Enrique Martínez-Carranza, Hugo Barajas, Luis-David Alcaraz, Luis Servín-González, Gabriel-Yaxal Ponce-Soto, Gloria Soberón-Chávez

**Affiliations:** ^1^Departamento de Biología Molecular y Biotecnología, Instituto de Investigaciones Biomédicas, Universidad Nacional Autónoma de México, Mexico City, Mexico; ^2^Departamento de Biología Celular, Facultad de Ciencias, Universidad Nacional Autónoma de México, Mexico City, Mexico; ^3^Laboratorio Nacional de Ciencias de la Sostenibilidad, Instituto de Ecología, Universidad Nacional Autónoma de México, Mexico City, Mexico; ^4^Departamento de Ecología Evolutiva, Instituto de Ecología, Universidad Nacional Autónoma de México, Mexico City, Mexico

**Keywords:** essential-genes, core-genome, non-orthologous gene displacement, *Escherichia coli*, whole genome sequence databases

## Abstract

The definition of bacterial essential genes has been widely pursued using different approaches. Their study has impacted several fields of research such as synthetic biology, the construction of bacteria with minimal chromosomes, the search for new antibiotic targets, or the design of strains with biotechnological applications. Bacterial genomes are mosaics that only share a small subset of gene-sequences (core genome) even among members of the same species. It has been reported that the presence of essential genes is highly variable between closely related bacteria and even among members of the same species, due to the phenomenon known as “non-orthologous gene displacement” that refers to the coding for an essential function by genes with no sequence homology due to horizontal gene transfer (HGT). The existence of dormant forms among bacteria and the high incidence of HGT have been proposed to be driving forces of bacterial evolution, and they might have a role in the low level of conservation of essential genes among related bacteria by non-orthologous gene displacement, but this correlation has not been recognized. The aim of this mini-review is to give a brief overview of the approaches that have been taken to define and study essential genes, and the implications of non-orthologous gene displacement in bacterial evolution, focusing mainly in the case of *Escherichia coli*. To this end, we reviewed the available literature, and we searched for the presence of the essential genes defined by mutagenesis in the genomes of the 63 best-sequenced *E. coli* genomes that are available in NCBI database. We could not document specific cases of non-orthologous gene displacement among the *E. coli* strains analyzed, but we found that the quality of the genome-sequences in the database is not enough to make accurate predictions about the conservation of essential-genes among members of this bacterial species.

## Variability of Essential Genes Among Bacteria: a Challenge to the Concept of the Core-Genome

The identification of the gene-products that are essential for the function of a living cell in a particular environment is a crucial task for the understanding of the molecular fundaments of life (Juhas et al., 2011), and the evolution of the different phylogenetic taxa (Charlebois and Doolittle, 2004). Thus the definition of these indispensable gene-products has been widely studied mainly using bacteria as model organisms, due to their relative simplicity and small genome size (Sleator, 2010; Juhas et al., 2011; Glass et al., 2017). In this mini-review we focus on genes that are defined as indispensable for bacteria growing in rich media, with controlled abiotic factors (i.e., temperature, agitation) which tend to be more conserved than non-essential genes (Ish-Am et al., 2015).

The definition of a bacterial species as an evolutionary coherent biological group is a non-realistic task since bacterial genomes are mosaics that contain segments of different phylogenetic origins (Mira et al., 2004; Fraser et al., 2009; Zhaxybayeva et al., 2009; González-Casanova et al., 2014). Also, bacterial genomes present an enormous genetic variability (Ciccarelli et al., 2006; Hug et al., 2016), and a high incidence of horizontal gene transfer (HGT) (Dagan et al., 2008; Darmoh and Leach, 2014; Soucy et al., 2015). These anomalies in the phylogenetic coherence of bacterial species led to the definition of the concepts of the core-, accessory-, and pan-genome.

The core-genome is defined as the set of genes shared by all the individuals that belong to a certain species (and therefore should contain the essential genes particular to that species); the accessory-genome consists of genes that are present only in a fraction of the members of a species, or even just in one individual. Finally, the pan-genome constitutes the complete genetic repertoire of a bacterial species, that is formed by the addition of the core-genome and accessory-genome (Medini et al., 2005; Tettelin et al., 2008; Guimarães et al., 2015). Most genes belonging to the accessory-genome are acquired by HGT, and are supposed to encode adaptive functions that are not essential for the biology of a particular species (Dagan et al., 2008; Darmoh and Leach, 2014; Soucy et al., 2015).

Core-genome genes have been postulated to represent the evolutionary coherent bacterial genetic information and have been used to construct phylogenetic trees (Ciccarelli et al., 2006; Hug et al., 2016). However, it has been reported that core-genome conserved genes like tRNA synthases (Soucy et al., 2015), and the transcriptional regulator CRP (Soberón-Chávez et al., 2017), showed phylogenetic inconsistencies in some bacteria, indicating that even conserved genes might be prone to be inherited by HGT. Furthermore, it has been shown that genes coding for essential functions for the biology of *Azotobacter vinelandii* have been inherited by HGT and thus form part of the accessory-genome of the Pseudomonads (the phylogenetic group that contains *Pseudomonas* and *Azotobacter* species) (González-Casanova et al., 2014).

The inconsistency between the definition of the core-genome and these anomalies in *A. vinelandii* taxonomy have been analyzed using as a framework the population genetics model proposed by Blath et al. (2013, 2015) that has been recently reviewed (Shoemaker and Lennon, 2018). The model proposed by Blath et al. (2013, 2015) states that organisms forming dormant structures such as spores and cysts that persist in the environment for periods much larger than their generation times, give rise to an ancestral genetic pool that temporally escapes from natural selection and other evolutionary forces. It has been postulated that bacteria fulfill the postulates of Blath’s model (González-Casanova et al., 2014), since many species form spores or cysts, and phages contain fragments of bacterial genomes that can be considered as forming part of dormant forms (Lang et al., 2012).

The use of comparative genomics for the study of essential genes assumes phylogenetic conservation among related organisms (Grazziotin et al., 2015; Luo et al., 2015; Oren et al., 2015), and hence that these genes form part of the core-genome (Tazzyman and Bonhoeffer, 2015; Zhang et al., 2015). However, the analysis of essential genes by comparative genomics has shown that only a small number of essential gene-sequences are conserved among different bacterial groups (Ciccarelli et al., 2006; Juhas et al., 2011; Bergmiller et al., 2012). For example, a detailed search for conserved essential genes among 147 prokaryotes (130 Bacteria and 17 Archaea) rendered only 35 conserved genes, most of which participate in transcription and translation (Charlebois and Doolittle, 2004). Furthermore, when the number of prokaryotic whole-genome sequences reached 1000 (930 Bacteria and 70 Archaea), it was reported that there were no common gene-sequence that were conserved in all of these organisms, and that only four genes (two coding for proteins and two for RNAs) were conserved among Bacteria (Lagesen et al., 2010). Furthermore, there have also been reports that essential gene-sequences can vary among strains belonging to the same bacterial species (Juhas et al., 2011; Turner et al., 2015; Ibberson et al., 2017).

The reduced number of conserved essential genes among different bacteria has generated the concept of non-orthologous gene displacement that describes a variant form of a pathway in which a certain essential gene is replaced by a functional equivalent with no DNA homology, that differs in its evolutionary origin (Koonin et al., 1996; Charlebois and Doolittle, 2004). To have a less stringent definition of a conserved essential gene, that contemplates the possibility that non-orthologous gene-displacement “erase” some of the sequence conservation of essential genes, the concept of persistent genes has been developed (Fang et al., 2005, 2008; Acevedo-Rocha et al., 2013; Luo et al., 2015).

The implications of the high incidence of non-orthologous gene displacement for the evolution of bacteria has not been addressed in the context of synthetic biology and of the study of essential genes, but is in accordance with the postulates of the Blath et al. (2013, 2015) probabilistic model in population genetics, and the high incidence of HGT.

## The Search for Bacteria With Reduced Genomes

The isolation of bacterial cells with reduced genomes is an active area of research for synthetic biology (Gil et al., 2004), that has shown to be important not only to define the indispensable genetic information required for a viable cell, but also for biotechnological purposes. This approach has been used to produce strains with suitable characteristics for industrial applications (Moya et al., 2009), and to define targets for the development of new antibiotics (Juhas et al., 2012a). The bacterial cell with the smallest genome that was chemically synthesized is *Mycoplasma mycoides* JCV1-Syn3.0 that contains a 531 kbp genome with a total of 473 genes (48 of them are not essential) (Hutchison et al., 2016; Glass et al., 2017).

In the case of *Escherichia coli* K-12, a strain with several deletions which eliminate 29.7% of its genome was constructed; this strain presents a lower growth rate, morphological changes, and multiple nucleoids (Hashimoto et al., 2005; Supplementary Table S1), while another deletion-derivative with the removal of 35% of its genome, produced fast-growing cultures with a higher cell-density and was engineered to produce increased threonine levels (Lee et al., 2009; Juhas et al., 2014). In addition, *E. coli* K-12 MG1655 derivatives with multiple deletions which eliminate around 15% of the genome, including all IS sequences and transposons, was reported to present a high growth rate and a high electroporation efficiency (Posfai et al., 2006; Supplementary Table S1).

**FIGURE 1:**
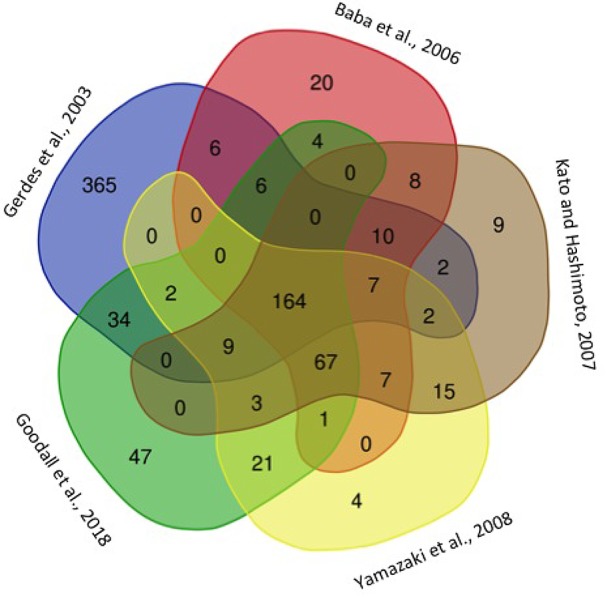
Venn diagram showing the *Escherichia coli* predicted essential genes in different studies. The name of the first author of each study where essential genes are predicted is shown. The total number of genes predicted in these studies is presented in Supplementary Table S1.

The definition of essential genes was originally pursued by a mutagenesis strategy. They were defined as those in which it was not possible to isolate mutants, to eliminate by deletion, or whose expression could not be silenced using antisense-RNAs (Rusmini et al., 2014). In the case of *E. coli* (Supplementary Table S1), the first attempt to isolate mutants proposed that 620 genes were essential (Gerdes et al., 2003), but the selection of specific deletion-mutants in each open reading frame (ORF) enabled Baba et al. (2006) to reduce this number to 303. Recently a saturated transposon mutagenesis and sequencing-strategy defined 358 essential genes in *E. coli* K-12 strain BW25113 (Goodall et al., 2018); 248 of these essential genes are shared with the Keio collection (Baba et al., 2006) and also with the PEC database (Yamazaki et al., 2008), while 47 genes defined as essential are particular to this saturated-mutagenesis strategy (Goodall et al., 2018; **Figure 1**).

Kato and Hashimoto (2007) reported 268 essential genes in *E. coli* based on the inability to isolate deletions that include any of these genes, and 152 essential genes were identified in this bacterium by the inability to be silenced by antisense-RNAs (Meng et al., 2012; Supplementary Table S1). The lower number of essential genes detected using antisense-RNAs silencing might be because this method still permits a low level of expression in contrast to the mutagenesis strategy. The comparison of the specific essential genes identified in some of these studies (**Figure 1**) shows that only 164 of the total of 4218 *E. coli* genes were identified as essential in all of them (Supplementary Table S2). This result implies that only around 3.8% of the *E. coli* genome encodes for essential functions. However, it seems likely that the 164 common essential genes are not enough to render a viable *E. coli* derivative, since this number is considerably smaller than the number of genes that have been identified in any of the reported approaches to identify essential genes in this bacterium. Therefore, it is possible that the small number of essential genes that are common in all these studies (**Figure 1** and Supplementary Table S2) are a product of the different experimental approaches taken and of the variability of culture conditions that were used.

Another approach for the definition of essential genes is through comparative genomics and gene conservation, looking for genes that have been reported to be essential in one organism, that are conserved among different bacteria (Arigoni et al., 1998; Glass et al., 2009; Alcaraz et al., 2010; Acevedo-Rocha et al., 2013). The use of comparative genomics in the search of essential genes (Luo et al., 2015) has led to the construction of essential genes databases (Ye et al., 2013; Luo et al., 2014; Peng et al., 2017), and to define essential genes in determined bacterial species, such as *Burkholderia cenocepacia* (Juhas et al., 2012b).

## Variability of Essential Genes in *E. coli*

The use of *E. coli* as a model organism has been crucial to the fields of molecular biology and synthetic biology (Juhas et al., 2011), so we decided to analyze with some detail the reported essential genes of this bacterium in the genome of different strains. Thus, to determine the existence of non-orthologous gene displacement among different *E. coli* strains we searched for the 303 essential genes defined in the Keio collection in 63 whole genome sequences. We chose these *E. coli* genomes because they are supposedly the best annotated genomes deposited in the NCBI database (the NCBI accession numbers of the whole genome sequences of these 63 *E. coli* strains are shown in Supplementary Table S3).

The retrieved information from the 63 genomes shows that only four of these genomes (W3110, MG1655, DH1, and BW2952) contained the 303 essential genes described in the Keio collection (see Supplementary Figure S1). We found that 11 genes were absent in more than one of the analyzed genomes and that another 11 genes were absent in only one of these genomes (Supplementary Figure S1 and **Table 1**). Most of the genes that are absent in more than one of the genomes analyzed encode for antitoxins or phage repressor proteins (**Table 1**). Thus it seems likely that these genes are essential in the BW25113 strain used to isolate the mutants of the Keio collection because this strain carries the corresponding toxin or phage that is counteracted by their products, while these toxins and phages seem to be absent in the genomes that lack these supposedly essential genes. Even two K-12 derived strains lack *cohE* that encodes for the e14 prophage repressor (**Table 1**). Also the *waaU* gene, involved in lipopolysaccharide (LPS) biosynthesis, is missing in several of the 63 strains; it is very much likely that strains that lack *waaU* have a different LPS structure than that of strain BW25113.

**Table 1:** Essential genes defined in the Keio collection that are absent in the 63 *Escherichia coli* whole genome sequences analyzed (refers to data shown in **Figure 1**).

		Number of
Gene		strain where the
name	Assigned function of gene product	gene is absent^∗^
*bcsB*	Cellulose biosynthesis	1 (HS)
*dxr*	Participates in isoprenoid synthesis, catalyzing the conversion of 1-deoxy-D-xylulose-5-phosphate (DXR) to 2-methyl-D-erythritol-4-phosphate (MEP)	1 (CFT073)
*dnaA*	Chromosomal replication initiator protein DnaA	1 (CFT073)
*infC*	Factor3 for the initiation of protein translation	1 (P12B)
*dnaX*	DNA polymerase III/DNA elongation factor III, tau and gamma subunits	1 (P12B)
*mukB*	Chromosome condensin MukBEF, ATPase and DNA-binding subunit	1 (P12B)
*rplE*	Ribosomal protein E of the large ribosome subunit	1 (P12B)
*serS*	Seryl-tRNA synthetase (SerRS)	1 (P12B)
*plsB*	Membrane-bound glycerol-3-phosphate acyltransferase catalyzes the first step in phospholipid biosynthesis	1 (536)
*secA*	Couples ATP hydrolysis with protein secretion through the cell membrane	1 (UM146)
*trmD*	SAM-dependent tRNA m(1)G37 methyltransferase	1 (APEC 01)
*chpS*	ChpS antitoxin; toxin is ChpB	6
*maze*	MazE antitoxin; toxin is MazF	7
*yelM*	YelM antitoxin; toxin is YoeB	15
*alsK*	D-allose kinase	21
*cohE*	Ci-like repressor of e14 prophage	45
*racR*	Rac prophage repressor	50
*waaU*	Lipopolysaccharide core biosynthesis	55
*mqsA*	MqsA antitoxin; toxin is MqsR	50
*yibJ*	Putative protein belonging to the “rearrangement hot spot” (RHS) family	24
*ydiB*	Qin prophage predicted protein	18
*dicA*	Transcriptional repressor of *dicB*, Qin prophage	24


*^∗^In the case where the gene is absent only in one strain, the name of the strain is shown in parenthesis. To perform this search, the encoded protein sequence of each of the 303 *E. coli* K-12 essential genes defined by Baba et al. (2006) was used as a query to perform local alignments (using TBLASTN) against the complete genome sequences of the 63 *E. coli* strains used in this study. In the case where one gene seemed to be absent, it was further searched in the corresponding K-12 locus to determine whether the whole sequence was missing, or whether it was present but having a partial deletion or frameshift mutation compared with the K-12 genome (all data were obtained from the NCBI database). Missing essential genes in single genomes were also searched based on the synteny of the *E. coli* chromosomes using the Artemis program (Carver et al., 2012).*

The *alsK* gene, coding for the allose kinase that is involved in the phosphorylation of the hexose allose is absent in 21 of these 63 genomes. It is difficult to understand why AlsK would be essential for *E. coli* cultured in rich medium. Furthermore, *alsK* deletion mutants constructed by the Datsenko and Wanner (2000) procedure have been reported in strain MG1655 (Xia et al., 2017), showing that AlsK is not an essential protein. Thus, it is clear that *alsK* is not an essential gene as was previously reported (Baba et al., 2006) and coincides with the recently reported results (Goodall et al., 2018).

The data presented in **Table 1** show that some genes are essential only in specific genomic backgrounds and that they do not necessarily encode for fundamental traits for all members of a bacterial species, *E. coli* in this case. Furthermore, these genes are part of the accessory genome by definition, since they are not present in all the *E. coli* strains, and most of them are inherited by HGT (they form part of phages and other mobile genetic elements). These genes represent examples of essential genes (at least in some genetic backgrounds such as the BW25113 strain) that do not form part of the core-genome and highlight the fact that essential genes and genes of the core-genome are not synonymous concepts.

In contrast, all the 11 genes that are absent from only one of the genomes encode for metabolic functions, and in several instances, the activity of the encoded protein plays a central role in a fundamental cell processes, like DNA replication or protein translation or secretion (**Table 1**).

The apparent absence of essential genes in any of the 63 *E. coli* genomes included in this review could be due to non-orthologous gene-displacement, or to sequencing or annotation mistakes as has been reported previously (Charlebois and Doolittle, 2004). Surprisingly, a detailed genome analysis showed that multiple missing essential genes (10) by BLAST search were indeed present, as evidenced by synteny analysis. The BLAST algorithm wast not able to detect the genes because of sequencing errors in the reported genome sequences resulted in frameshifts that resulted in apparent absence of the genes (see Supplementary Figure S2).

The only case where there is a real deletion of any of the essential genes defined by Baba et al. (2006) is the *bcsB* gene (**Table 1**), which forms part of the *bcs* operon that encodes proteins involved in the synthesis of cellulose. The *bcsB* gene is absent as part of a deletion spanning 12 kb of the chromosome of the commensal HS strain (Rasko et al., 2008) which comprises most of the genes of the *bcs* operon. The *bcsB* gene was not found to be essential in the recently reported saturated transposon and sequencing strategy (Goodall et al., 2018).

The high frequency of sequencing errors in these 63 *E. coli* whole genomes shows that the majority of genome sequences that are available in databases are prone for false negatives when searching them with sequence homology tools in the comparative genomics studies of essential genes.

The definition of the core-genome that relies on homologous sequences that are present in all individuals of a particular species cannot be achieved if a high frequency of sequencing and annotation mistakes is populating databases. This sequence genome quality problem represents a relevant drawback in the study of essential genes using the available databases constructed for this purpose (Ye et al., 2013; Luo et al., 2014; Peng et al., 2017), and is an obstacle to evaluate the extent of non-orthologous gene displacement that has occurred in bacterial genomes.

## Concluding Remarks

The aim of this review is to address the high variability of essential bacterial genes in relation to bacterial evolution, and the role that comparative genomics plays in this field (Arigoni et al., 1998; Glass et al., 2009; Alcaraz et al., 2010; Acevedo-Rocha et al., 2013). We highlight the fact that the sequence conservation of essential genes among different phylogenetic groups of bacteria, and even among the same bacterial species, has been reported to be very low (Charlebois and Doolittle, 2004; Lagesen et al., 2010; Juhas et al., 2011).

The low level of sequence conservation among essential genes has been explained by the existence of a phenomenon called non-orthologous gene displacement that refers to the natural selection of essential functions encoded by genes with no sequence homology. These non-orthologous essential genes hence will not belong to the core-genome, which is the genetic information that has been used to build all bacterial taxonomy. To determine the extent of non-orthologous gene displacement that occurs in different bacterial species is a matter of great importance for the field of bacterial evolution.

In this mini-review we addressed the situation of the 303 essential *E. coli* genes defined by Baba et al. (2006), trying to determine whether non-orthologous gene displacement could be documented in this bacterial species. We show that the essentiality of some genes depends on the presence of other non-essential genes (like antitoxin and phage repressor genes), and that this is an important source of variability for the presence of essential genes. Another important source of variation is due to sequencing mistakes that make the essential genes invisible to commonly used bioinformatics tools (Supplementary Figure S2). The problem of sequencing and annotation mistakes in the use of comparative genomics for the study of essential genes has been reported previously (Charlebois and Doolittle, 2004). No case of non-orthologous gene displacement was documented.

It is a challenge for the research in the field of essential genes to identify the phylogenetic origin of essential genes that are encoded by non-orthologous sequences. To perform reliable comparative genomics analysis it is imperative to improve databases aiming for high quality sequence of genomes and assemblies so it is possible to identify interesting features like non-orthologous gene displacement.

## Author Contributions

EM-C made a bibliographic search for articles related to the theme of this mini-review and constructed **Figure 1**. GS-C conceived and designed the work. L-DA, G-YP-S, EM-C, and HB performed the database searches. GS-C, LS-G, L-DA, G-YP-S, and HB made substantial contributions for the analysis and interpretation of the information. GS-C, LS-G, EM-C, L-DA, and HB participated in drafting the work and revised it critically. GS-C, LS-G, EM-C, L-DA, G-YP-S, and HB approved the final version of the work, and agreed to be accountable for its content.

## Conflict of Interest Statement

The authors declare that the research was conducted in the absence of any commercial or financial relationships that could be construed as a potential conflict of interest.
