# Corrigendum: Variability of Bacterial Essential Genes Among Closely Related Bacteria: The Case of *Escherichia coli*

**DOI:** 10.3389/fmicb.2018.02330

**Published:** 2018-09-28

**Authors:** Enrique Martínez-Carranza, Hugo Barajas, Luis-David Alcaraz, Luis Servín-González, Gabriel-Yaxal Ponce-Soto, Gloria Soberón-Chávez

**Affiliations:** ^1^Departamento de Biología Molecular y Biotecnología, Instituto de Investigaciones Biomédicas, Universidad Nacional Autónoma de México, Mexico City, Mexico; ^2^Departamento de Biología Celular, Facultad de Ciencias, Universidad Nacional Autónoma de México, Mexico City, Mexico; ^3^Laboratorio Nacional de Ciencias de la Sostenibilidad, Instituto de Ecología, Universidad Nacional Autónoma de México, Mexico City, Mexico; ^4^Departamento de Ecología Evolutiva, Instituto de Ecología, Universidad Nacional Autónoma de México, Mexico City, Mexico

**Keywords:** essential-genes, core-genome, non-orthologous gene displacement, *Escherichia coli*, whole genome sequence databases

In the published article, there was an error regarding the affiliation for Luis David Alcaraz. As well as having affiliations 2, they should also have 3. The authors apologize for this error and state that this does not change the scientific conclusions of the article in any way.

The original article has been updated.

## Conflict of interest statement

The authors declare that the research was conducted in the absence of any commercial or financial relationships that could be construed as a potential conflict of interest.

