# Supplementary material for: Variability of Bacterial Essential Genes Among Closely Related Bacteria: The Case of Escherichia coli
Source: Front Microbiol. 2018 May 29;9:1059. doi: 10.3389/fmicb.2018.01059 (PMC5992433; doi:10.3389/fmicb.2018.01059)
Supplement: Supplementary file 1 [file Presentation_1.PDF]

Table S1. *Escherichia coli* essential genes identified by different approaches

E. coli K12 strain	# essential genes identified	Identification strategy	Comments	Reference
MG1655	620	Transposon mutagenesis	Mutations in 3,746 genes (87%) were selected.	Gerdes et al., 2003.
BW25113	303	Single gene deletion, using the methodology designed by Datsenko and Wanner, 2000.	The systematic deletion of each open reading frame (ORF) lead to the construction of the Keio collection, which contains deletions in all the *E. coli* ORFs, with the exception of the 303 genes that were defined as essential.	Baba et al., 2006.
MG1655	Undefined	Large scale deletions that include 29. 7% of the chromosome.	The *E. coli* derivative with the larger deletion has a lower growth rate, modified morphology and multiple small nucleoids	Hashimoto et al., 2005
MG1655	268	Large scale deletions	The derivative with the larger deletion reported previously (Hashimoto et al., 2005) was used to define genes that were essential using mini-F' plasmids to complement specific deletion mutants.	Kato and Hashimoto, 2007.
MG1655	Undefined	Construction of several derivatives called "multiple deletion series" (MSD) with up to 15% of genome reduction	MSD42 with 14.3% chromosome deletion (704 genes, including insertion sequences and transposons) has a high electrophoretic ability and good growth profile.	Posfai et al, 2006.
Several strains	300	Online profiling of the *E. coli* genome database (PEC database)	The essentiality data of genes included in this database are based on the compilation from published single-gene essentiality studies and on cell growth studies of large-deletion mutants.	Yamazaki et al, 2008.
MG1655	152	Genes unable to be silenced by antisense-RNAs	The lower number of essential genes identified might be due to the low,	Meng et al., 2012.

			but significant expression of silenced genes.	
BW25113	358	Transposon-directed insertion site sequencing (TraDIS)	Through this analysis new essential genes and genes incorrectly designated as essential were identified; 248 essential genes overlap with the Keio collection and the PEC database.	Goodall et al., 2018.

Table S2. List of *E. coli* genes identified as essential in the studies included in figure 1

Study (Analysis)	Number of shared genes	Shared genes
Baba et al., 2006. Gerdes et al., 2003. Goodall et al., 2018. Kato and Hashimoto, 2007. Yamazaki et al, 2008.	164	gmk dapE murE mreB valS pth yqgF lspA glmU trmD def rpoB pgk holA dapD rplW folA hemG tyrS ispA folE parE kdsB rplO mrdA lolD pssA nrdA fabG dnaB rplF map argS rplN rplT rpoC lpxA ftsA glmS rpoH tmk mrdB glnS lolE serS rpsN frr cysS hemH secY mukF dnaA gapA ispE thrS ispD rpsP rpsB lpxB dfp fbaA topA leuS lepB zipA ftsW murI secA rpsJ era msbA thiL gyrA rpsQ rpsM aspS ribF ligA ftsY rpsE ftsI grpE ribE hisS dut adk pyrG fmt tsf proS fusA trpS rpsL lolC fabB ribD metG rplD pyrH asnS mreC coaD suhB rplJ plsC hemC pgsA fabD rpsS murG hemL dnaE lgt ispF fabA cca murC dxr holB fabI rplC ppa hemB acpS mukB gltX rplS ftsL rplB ispB glyQ rplM ftsQ fabZ lpxH dapA lpxK nadE infB ispH rpsI dapB metK birA murD rplX dxs rpsH dnaX rplL rplR accA mukE ffh nrdB dnaC ispG ftsZ groS hemD ribA cdsA infC asd
Baba et al., 2006. Gerdes et al., 2003. Kato and Hashimoto, 2007. Yamazaki et al, 2008.	7	secD rne secM ftsE ribB secF folk
Gerdes et al., 2003. Goodall et al., 2018. Kato and Hashimoto, 2007. Yamazaki et al, 2008.	9	nusB ileS dnaG dnaT rpoD alaS glyS parC coaA
Baba et al., 2006. Goodall et al., 2018. Kato and Hashimoto, 2007. Yamazaki et al, 2008.	67	ispU ubiA nusA can mraY pheS rpsK orn fldA accC nadD mreD rpmC tilS gpsA rpsA rpmB acpP yidC accB psd lpxC rplV rplP rpmD hemA obgE yrfF lnt rpmH der lolB ftsB rpsC gyrB ssb rpsD prfA kdsA lpxD rpsG accD ftsH rpsR murB murA rplQ ribC nusG rplU folD lexA lolA murF csrA pheT folC eno yihA dnaN rpmA secE infA rplE rpoA plsB rnpA
Baba et al., 2006. Gerdes et al., 2003. Kato and Hashimoto, 2007.	10	yaeT prsA kdtA yeaZ yjeE yjgP rlpB yjgQ mviN imp
Baba et al., 2006. Gerdes et al., 2003. Goodall et al., 2018.	6	ubiB wzyE racR cydC cydA dicA
Gerdes et al., 2003. Kato and Hashimoto, 2007. Yamazaki et al, 2008.	2	efp priA
Gerdes et al., 2003. Goodall et al., 2018. Yamazaki et al, 2008.	2	coaE groL
Baba et al., 2006. Kato and Hashimoto, 2007. Yamazaki et al, 2008.	7	degS yceQ ftsK ftsX yejM ftsN spot
Baba et al., 2006. Goodall et al., 2018. Yamazaki et al, 2008.	1	prmC
Goodall et al., 2018. Kato and Hashimoto, 2007. Yamazaki et al, 2008.	3	rho hda prfB
Baba et al., 2006. Gerdes et al., 2003.	6	minD ymfK rnc chpS yagG ygiT
Gerdes et al., 2003. Kato and Hashimoto, 2007.	2	hemK yhbG
Gerdes et al., 2003. Goodall et al., 2018.	34	yciS tktA fabH ubiX rpe rluD sucA thyA iscS ubiH ybeY dapF ydaS aceF hscA rimM ydhL priB ubiE rplY rpsF cydD cydB yjbS glyA ubiG rpsT rpsO hemE rnt sucB fdx yffS tonB

Kato and Hashimoto, 2007. Yamazaki et al, 2008.	15	trpT thrU cysT argX argU leuU serV rnpB glyT hisR ffs leuZ proM leuW serT
Goodall et al., 2018. Yamazaki et al, 2008.	21	glmM tsaB rsgA bamA lptE prs bamD lptD tsaE lptF murJ tsaC nadK lptG lptA mnmA folB tsaD erpA lptB waaA
Gerdes et al., 2003.	365	cvpA ydcQ ybjP ynfD yfiD gcvR hscB csgE yddE seqA yejE hyaA yagH yneG argR yadI ybbC ydgA rhaD ybeA insE3 ruvC tdcR yncA yebE ynfA mog pdxJ cmk yibG rnhB yjbM yaiS yjgF relE yhbS yggL yegL hybD ypjF aroH eutN yqjA ydjA yhcK hns mraW yffO trg elbA ynaJ yadF insE1 ybgP ydgR appY ycdL yidP cheW mtn yebC yagV ygjQ paaX corA hnr yliG yjeO yacC yccD argC ydaL ymfI mglB rstB motA yhhM yedE yheN potD ydhD ycfP paaC yaiY rpoN essQ ydcZ nrdI ycdK poxA ymcD yebG trmE trxC dcm yfaD ychH ycbK ybaN yggA hyaC selD celC yciL xerC yagS rusA holC degQ yebY yhbQ ybjK mgsA ydiR ydcN ytfS oppC potC yehD ispZ yeeV yeiL yhbP lpdA ackA yfgE yfaV ynjD yccU yhbC insB4 nifU ybjQ ycjF glnP ychF ydaU ycgM tus aroK ycdX yhfQ yqjK ygfY yhbZ cysW slyD hycF trmU yfjG ydiO ymfJ tdk yfjF hcaC zraP rimI ygiZ hfq btuR uppS paaY ydhX ybcV miaA ygbA elaA phrB yhhL galF wzb ybfN yeaN mesJ ygbQ ychQ oppD ydiI tdcG menE yohL ycaC ycgS secB hslJ gor slp yaiN arcC ycfD ycdW ygaP tufB yncE yqfB yeiM engB tyrB torR ygdK torD yjbA ybhA ybaP ybjS insH5 yehC astB nudB yhcO ydhR acpD sgcB ybiA yqhA yecG ydfV atpC yhhF ydcV yheM yiiU araG yciK rnhA ybjC pgpA ptsN ddlB mntR yciA pyrF eutL ypdH yahH uidR yecP apt yeaO ndk gltL ypjC rimL agaV yeiJ ybhN tufA sgcA yfhC fepB rsd msyB purU engA ybaA yeeI yddK ydiT glnD nrdH rluA yhcF ydiY cynS cheR marA ybjR yjbL ybaV gatD ygbK ygfM cyoD pspE yddM ybcO araF ybaQ ydiZ atpF ybcN yafE dadA ppnK ynjA ybfC ribH ydaT ydjQ yqjB yjbO lplA yddQ ycdZ ydeE ynfG ybhQ yljA yegP cutC yffB agaW rep ydcU proC hyaD yfdX mscL insE4 insE5 rfaC yahF gltK yieL yqgE ydfR nhoA astA ycgX yjgJ cysE ansB dnaK dnaQ pnp pspC ygfB fliL galU ykfI fliJ ccmE ybfB ydjR gshB fis sufA btuE yfcH yoaB ycjI allA yffM gyrI ygaU yibA zwf ytfI crcB dsbA hybG yqiB hcaF yheL ybgC yraR ppiB ydgE ydcT ylbF yjjV celA hycI priC fliN
Baba et al., 2006.	20	ydfB chpR yefM yabQ yrbB yigP yraL tnaB alsK yqgD tdcF yhbV yibJ minE bcsB ydiL rfaK entD yhhQ yafF
Kato and Hashimoto, 2007.	9	yjeQ groEL yrbI ycfB oriC ygiG yfjA yacE mrsA
Yamazaki et al, 2008.	4	lptC polA kdsC rseP
Goodall et al., 2018.	47	yncH trpL rpmI lysS folP ygfZ yqeL ykfM lipA ycaR ydaE ygeF iraM dcd ynbG cydX pheM yddL relB ihfA ymfE ygeN yqcG iscU rpmF yedN safA yobI ymiB rpsU tusE guaA lpd rbfA cohE ptsI pdxH ygeG higA ttcC rplA lpxL hipB ydcD holD ydfO rplK

Table S3. Genome accession numbers of *Escherichia coli* strains analyzed in this work.

Strain	Accession number
Escherichia coli 042 uid161985	NC_017626, NC_017627
Escherichia coli 536 uid58531	NC_008253
Escherichia coli 55989 uid59383	NC_011748
Escherichia coli ABU 83972 uid161975	NC_017629, NC_017631
Escherichia coli APEC O1 uid58623	NC_008563, NC_009837, NC_009838
Escherichia coli APEC O78 uid187277	NC_020163
Escherichia coli ATCC 8739 uid58783	NC_010468
Escherichia coli BL21 DE3 uid161947	NC_012971
Escherichia coli BL21 DE3 uid161949	NC_012892
Escherichia coli BL21 Gold DE3 pLysS AG uid59245	NC_012947
Escherichia coli B REL606 uid58803	NC_012967
Escherichia coli BW2952 uid59391	NC_012759
Escherichia coli CFT073 uid57915	NC_004431
Escherichia coli clone D i14 uid162049	NC_017652
Escherichia coli clone D i2 uid162047	NC_017651
Escherichia coli DH1 uid161951	NC_017625
Escherichia coli DH1 uid162051	NC_017638
Escherichia coli E24377A uid58395	NC_009786, NC_009788, NC_009790, NC_009801
Escherichia coli ED1a uid59379	NC_011745, NC_009787, NC_009789, NC_009791
Escherichia coli ETEC H10407 uid161993	NC_017633, NC_017721, NC_017722, NC_017723, NC_017724
Escherichia coli HS uid58393	NC_009800
Escherichia coli IAI1 uid59377	NC_011741
Escherichia coli IAI39 uid59381	NC_011750
Escherichia coli IHE3034 uid162007	NC_017628
Escherichia coli JJ1886 uid226103	NC_022648, NC_022650, NC_022661
Escherichia coli K 12 substr DH10B uid58979	NC_010473, NC_022649, NC_022651, NC_022662
Escherichia coli K 12 substr MDS42 uid193705	NC_020518
Escherichia coli K 12 substr MG1655 uid57779	NC_000913
Escherichia coli K 12 substr W3110 uid161931	NC_007779
Escherichia coli KO11FL uid162099	NC_017660, NC_017661
Escherichia coli KO11FL uid52593	NC_016902, NC_016903, NC_016904
Escherichia coli LF82 uid161965	NC_011993
Escherichia coli LY180 uid219461	NC_022364
Escherichia coli NA114 uid162139	NC_017644
Escherichia coli O103 H2 12009 uid41013	NC_013353, NC_013354
Escherichia coli O104 H4 2009EL 2050 uid175905	NC_018650, NC_018651, NC_018652, NC_018654
Escherichia coli O104 H4 2009EL 2071 uid176128	NC_018661, NC_018662,

	NC_018663
Escherichia coli O104 H4 2011C 3493 uid176127	NC_018658, NC_018659, NC_018660, NC_018666
Escherichia coli O111 H 11128 uid41023	NC_013364, NC_013366, NC_013368, NC_013365, NC_013367, NC_013370
Escherichia coli O127 H6 E2348 69 uid59343	NC_011601, NC_011602, NC_011603
Escherichia coli O157 H7 EC4115 uid59091	NC_011350, NC_011351, NC_011353
Escherichia coli O157 H7 EDL933 uid57831	NC_002655, NC_007414
Escherichia coli O157 H7 TW14359 uid59235	NC_013008, NC_013010
Escherichia coli O157 H7 uid57781	NC_002127, NC_002128, NC_002695
Escherichia coli O26 H11 11368 uid41021	NC_013361, NC_013362, NC_013363, NC_013369, NC_014543
Escherichia coli O55 H7 CB9615 uid46655	NC_013941, NC_013942
Escherichia coli O55 H7 RM12579 uid162153	NC_017653, NC_017655, NC_017657, NC_017654, NC_017656, NC_017658
Escherichia coli O7 K1 CE10 uid162115	NC_017646, NC_017647, NC_017648, NC_017649, NC_017650
Escherichia coli O83 H1 NRG 857C uid161987	NC_017634, NC_017659
Escherichia coli P12b uid162061	NC_017663
Escherichia coli PMV 1 uid219679	NC_022370, NC_022371
Escherichia coli S88 uid62979	NC_011742, NC_011747
Escherichia coli SE11 uid59425	NC_011407, NC_011411, NC_011415, NC_011419, NC_011408, NC_011413, NC_011416
Escherichia coli SE15 uid161939	NC_013654, NC_013655
Escherichia coli SMS 3 5 uid58919	NC_010485, NC_010486, NC_010487, NC_010488, NC_010498
Escherichia coli UM146 uid162043	NC_017630, NC_017632
Escherichia coli UMN026 uid62981	NC_011739, NC_011749, NC_011751
Escherichia coli UMNK88 uid161991	NC_017639, NC_017641, NC_017643, NC_017640, NC_017642, NC_017645
Escherichia coli UTI89 uid58541	NC_007941, NC_007946
Escherichia coli W uid162011	NC_017635, NC_017636, NC_017637
Escherichia coli W uid162101	NC_017662, NC_017664, NC_017665
Escherichia coli Xuzhou21 uid163995	NC_017903, NC_017906, NC_017907

Figure S1. Heatmap showing the number of copies of the 303 essential genes defined in the Keio collection (Baba et al., 2006) in the 63 whole genome sequences of *E. coli* strains shown in Table S3. A) Heatmap of the 303 essential genes with a frame showing the genes that contain more or less than one copy. B) Close-up of the region of the heatmap framed in A, where genes are present in more or less than one copy in the genomes of the 63 *E. coli* strains analyzed.

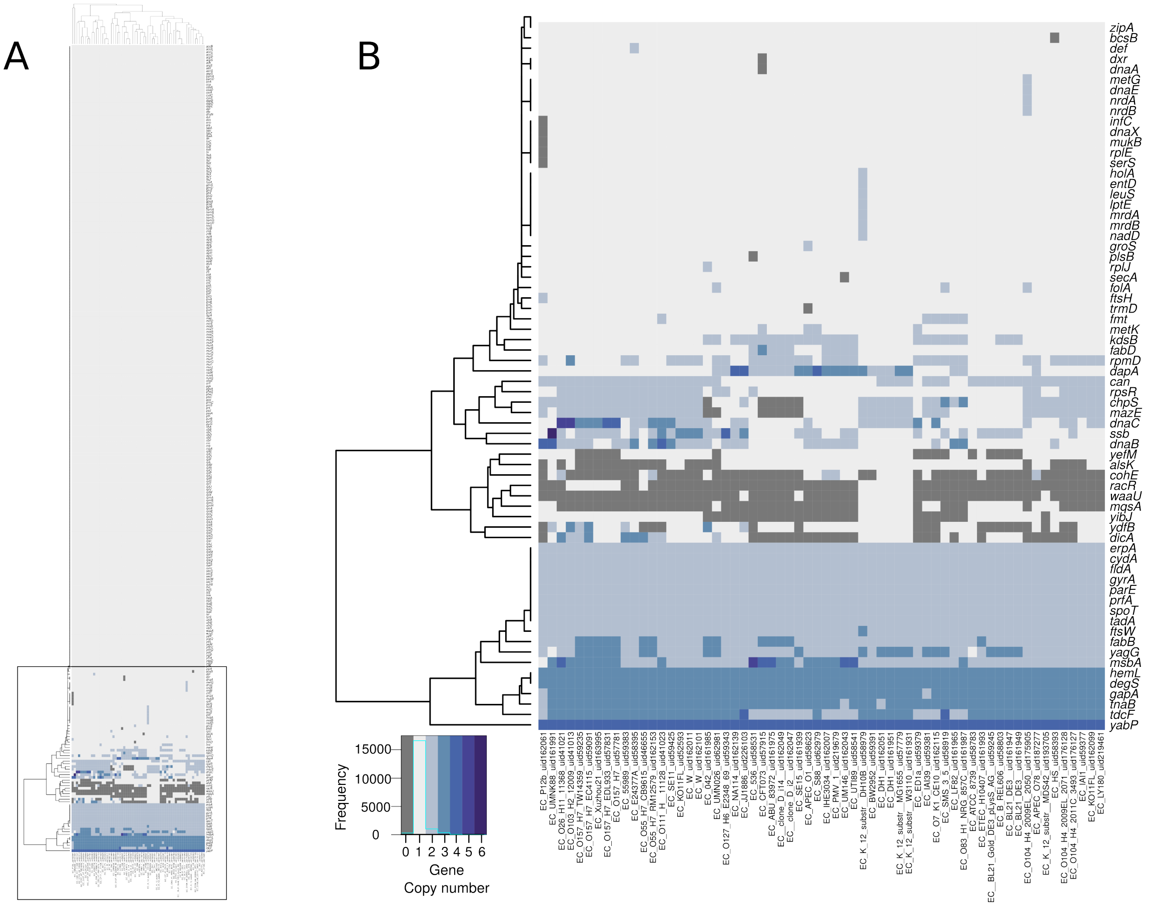

Figure S2. Reported gene sequences and their products of some essential genes that seemed to be absent from the analyzed genomes, and the theoretically "corrected" sequences. The nucleotides with the red square correspond to the presumed sequencing mistakes.

```
>C_RS21810 Escherichia coli CFT073 4384062:4385466 reverse, dnaA
gtgtcactttcgctttggcagcagtgtcttgcccgattgcaggatgagttaccagccaca
gaattcagtatgtggatacgcccattgcaggcggaactgagcgataacacgctggccctg
tacgcgccaaaccgttttgtcctcgattgggtacgggacaagtaccttaataatatcaat
ggactgctaaccagtttctgcggagcggatgccccacagctgcgttttgaagtcggcacc
aaaccggtgacgcaaacgccacaagcggcagtgacgagcaacgtcgcggcccctgcacag
gtggcgcaaacgcagccgcaacgtgctgcgccttctacgcgctcgggttgggataacgtc
ccggctccggcagaaccgacctatcgttctaacgtaaacgtcaaacacacgtttgataac
ttcgttgaaggtaaatctaaccaactggcgcgcgcggcggctcgccaggtggcagataac
cctggcggtgcttataacccgttgttcctttatggcggcacgggtttgggtaaaactcac
ctgctgcatgcggtgggtaacggcattatggcgcgcaagccgaatgccaaagtggtttat
atgcactccgagcgctttgttcaggacatggttaaagccctgcaaaacaacgcgatcgaa
gagtttaaacgctactaccgttccgtagatgcacgtgctgatcgacgatattcagtttt
tgctaataaagaacgatctcaggaagagtttttccacaccttcaacgccctgctggaagg
taatcaacagatcattctcacctccgatcgctatccgaaagagatcaacggcgttgagga
tcgtttgaaatcccgcttcggctggggactgactgtggcgatcgaaccgccagagctgga
aacccgcgtggcgatcctgatgaaaaaggccgacgaaaacgacattcgtttgccgggcga
agtggcgttctttatcgccaagcgtctacgatctaacgtacgtgagttggaaggcgcgct
gaaccgcgtcattgccaacgccaactttaccggacgggcgatcaccatcgacttcgtgcg
tgaggcgctgcgtgacttgctggcattgcaggaaaaactggtcaccatcgacaatattca
gaagacggtggcggagtactacaagatcaaagtcgcggatctcctttccaagcgtcgatc
ccgctcggtggcccgtccgcgccagatggcgatggcgctggcgaaagagctgactaacca
cagtcttccggagattggcgatgcctttggtggtcgtgaccacacgacggtgcttcatgc
ctgccggaagatcgagcagttgcgtgaagagagccacgatatcaaagaagatttttctaa
tttaatcagaacattgtcatcgtaa
```

DnaA protein reported sequence

```
>c4385466-4384062_1 Escherichia coli CFT073, DnaA
VSLSLWQQCLARLQDELPATEFSMWIRPLQAELSDNTLALYAPNRFVLDWVRDKYLNNIN
GLLTSFCGADAPQLRFEVGTKPVTQTPQAAVTSNVAAPAQVAQTQPQRAAPSTRSGWDNV
PAPAEPTYRSNVNVKHTFDNFVEGKSNQLARAAARQVADNPGGAYNPLFLYGGTGLGKTH
LLHAVGNGIMARKPNAKVVYMHSERFVQDMVKALQNNAIEEFKRYYRSVDARADRRYSVF
C**RTISGRVFPHLQRPAGR*STDHSHLRSLSERDQRR*GSFEIPLRLGTDCGDRTARAG
NPRGDPDEKGRRKRHSFAGRSGVLYRQASTI*RT*VGRRAEPRHCQRQLYRTGDHHRLRA
*GAA*LAGIAGKTGHHRQYSEDGGGVLQDQSRGSPFQASIPLGGPSAPDGDGAGERAD*P
QSSGDWRCLWWS*PHDGASCLPEDRAVA*REPRYQRRFF*FNQNIVIVX
```

DnaA protein verified sequence

```
>c4385466-4384062_1 Escherichia coli CFT073, DnaA
VSLSLWQQCLARLQDELPATEFSMWIRPLQAELSDNTLALYAPNRFVLDWVRDKYLNNIN
GLLTSFCGADAPQLRFEVGTKPVTQTPQAAVTSNVAAPAQVAQTQPQRAAPSTRSGWDNV
PAPAEPTYRSNVNVKHTFDNFVEGKSNQLARAAARQVADNPGGAYNPLFLYGGTGLGKTH
LLHAVGNGIMARKPNAKVVYMHSERFVQDMVKALQNNAIEEFKRYYRSVDALLIDDIQFF
ANKERSQEEFFHTFNALLEGNQQIILTSDRYPKEINGVEDRLKSRFGWGLTVAIEPPELE
TRVAILMKKADENDIRLPGEVAFFIAKRLRSNVRELEGALNRVIANANFTGRAITIDFVR
EALRDLLALQEKLVTIDNIQKTVAEYYKIKVADLLSKRRSRSVARPRQMAMALAKELTNH
SLPEIGDAFGGRDHTTVLHACRKIEQLREESHDIKEDFSNLIRTLSS*
```

```
>C_RS00965 Escherichia coli CFT073 203554:204752 forward, dxr
atgaagcaactcaccattctgggctcgaccggctcgattggttgcagcacgctggacgtg
gtgcgccataatcccgaacacttccgcgtagttgcgctggtggcaggcaaaaatgtcact
cgcatggtagaacagtgcctggaattctctccccgctatgccgtaatggacgatgaagcg
agtgcgaaacttcttaaaatgatgctacagcaacaggggagccgcaccgaagtcttaagt
gggcaacaagccgcttgcgatatggctgcgcttgaggatgttgatcaggtgatggcagcc
attt gttggcgctgctgggctgttacctacgcttgctgcgatccgcgcgggtaaaaccat
tttgctggccaataaagaatcactggttacctgcggacgtctgtttatggacgccgtaaa
gcagagcaaagcgcaattgttaccggtcgatagcgaacataacngctatttttcagagtt
taccgcaacctatccagcataatctgggatacgctgaccttgagcaaaatggcgtggtgt
ccattttacttaccgggtctggtggccctttccgtgagacgccattgcgcgatttggcaa
caatgacgcccgatcaagcctgccgtcatccgaactggtcgatgggacgtaaaatttccg
tcgattcggctaccatgatgaacaaaggtctggaatacattgaagctcgttggctgttta
acgccagcgccagccagatggaagtgctgattcacccgcagtcagtgattcactcaatgg
tgcgctaccaggacggcagtgttctggcgcagctgggtgaaccggatatgcgtacgccaa
ttgctcacaccatggcatggccgaatcgcgtgaactctggcgtgaagccgctcgattttt
gcaaactaagtgcgttgacatttgccgcaccggattatgatcgttatccatgtctgaaac
tggcgatggaggcgtttgaacaaggccaggcagcgacgacagcattgaatgccgcaaatg
aaatcaccgtggctgcttttcttgcgcaacaaatccgctttacggatatcgctgcgttga
atttatccgtactggaaaaaatggatatgcgcgaaccacaatgtgtggatgatgtgttat
ctgttgatgcgaacgcgcgtgaagtcgccagaaaagaggtgatgcgtctcgcaagctga
```

Dxr protein reported sequence

```
>C_RS00965 Escherichia coli CFT073 203554:204752, Dxr
MKQLTILGSTGSIGCSTLDVVRHNPEHFRVVALVAGKNVTRMVEQCLEFSPRYAVMDDEA
SAKLLKMMLQQQGSRTEVLSGQQAACDMAALEDVDQVMAAICWRCWAVTYACCDPRG#NH
FAGQ#RITGYLRTSVYGRRKAEQSAIVTGR+RT#XLFFRVYRNLSSIIWDTLTLSKMAWC
PFYLPGLVALSVRRHCAIWQQ*RPIKPAVIRTGRWDVKFPSIRLP**TKVWNTLKLVGCL
TPAPARWKC*FTRSQ*FTQWCATRTAVFWRSWVNRICVRQLLTPWHGRIA*TLA*SRSIF
AN#VR*HLPHRIMIVIHV*NWRWRRLNKARQRRQH*MPQMKSPWLLFLRNKSALRISLR*
IYPYWKKWICANHNVWMMCYLLMRTRVKSPEKR*CVSQA
```

Dxr protein verified sequence

```
>C_RS00965 Escherichia coli CFT073 203554:204752, Dxr
MKQLTILGSTGSIGCSTLDVVRHNPEHFRVVALVAGKNVTRMVEQCLEFSPRYAVMDDEA
SAKLLKMMLQQQGSRTEVLSGQQAACDMAALEDVDQVMAAIVGAAGLLPTLAAIRAGKTI
LLANKESLVTCGRLFMDAVKQSKAQLLPVDSEHNAIFQSLPQPIQHNLGYADLEQNGVVS
ILLTGSGGPFRETPLRDLATMTPDQACRHPNWSMGRKISVDSATMMNKGLEYIEARWLFN
ASASQMEVLIHPQSVIHSMVRYQDGSVLAQLGEPDMRTPIAHTMAWPNRVNSGVKPLDFC
KLSALTFAAPDYDRYPCLKLAMEAFEQGQAATTALNAANEITVAAFLAQQIRFTDIAALN
LSVLEKMDMREPQCVDDVLSVDANAREVARKEVMRLAS*
```

```
>P12B_RS02530 Escherichia coli P12b 512225:514157, dnaX
atgagttatcaggtcttagcccgaaaatggcgcccacaaacctttgctgacgtcgtcggc
caggaacatgtgctgaccgcactggcgaacggcttgtcgttagggcgtattcatcatgct
tatcttttttccggcacccgtggcgtcggaaaaacctctatcgcccgactgctggcgaag
gggctaaactgcgaaaccggcattaccgcgacgccgtgcggcgtgtgcgataactgtcgt
gaaatcgagcaggggcgctttgtcgatctgattgaaatcgacgccgcctcgcgcaccaaa
gttgaagatacccgcgacctgctggataacgtccagtacgctccggcgcgtggtcgtttc
aaagtttatctgatcgacgaagtgcatatgctgtcgcgccacagctttaacgcactgtta
aaaacccttgaagagccgccggagcacgttaagtttctgctggcgacgaccgatccacag
aaattgccggtgacgattttgtcacgctgtctgcaatttcatctcaaggcgctggatgtc
gagcaaattcgccatcagcttgagcacatcctcaacgaagaacatatcgctcacgagccg
cgggcgctgcaattgctggcacgcgccgctgaaggcagcctgcgagatgccttaagtctg
accgaccaggcgattgccagcggtgacggccaggtttcaacccaggcggtcagtgcgatg
ctgggtacgcttgacgacgatcaggcgctgtcgctggttgaagcgatggtcgaggccaac
ggcgagcgcgtaatggcgctgattaatgaagccgctgcccgtggtatcgagtgggaagcg
ttgctggtggaaatgctcggcctgttgcatcgtattgcgatggtacaactttcgcctgct
gcacttggcaacgacatggccgccatcgagctgcggatgcgtgaactggcgcgcaccata
ccgccgacggatattcagctttactatcagacgctgttgattggtcgcaaagaattaccg
tatgcgccggaccgtcgcatgggcgttgagatgacgctgctgcgcgcgctggcattccat
ccgcgtatgccgctgcctgagccagaagtgccacgacagtcctttgcacccgtcgcgcca
acggcagtaatgacgccaacccaggtgccgccgcaaccgcaatcagcgccgcagcaggca
ccgactgtaccgctcccggaaaccaccagccaggtgctggcggcgcgccagcagttgcag
cgcgtgcagggagcaaccaaagacaaaaaagagtgaaccggcagccgctacccgcgcgcg
gccggtgaataacgctgcgctggaaagactggcttcggtcaccgatcgcgttcaggcgcg
tccggtgccatcggcgctggaaaaagcgccagccaaaaaagaagcgtatcgctggaaggc
gaccactccggtgatgcagcaaaaagaagtggtcgccacgccgaaggcgctgaaaaaagc
gctggaacatgaaaaaacgccggaactggcggcgaagctagcggcagaagccattgagcg
cgacccgtgggcggcacaggtgagccaactttcgctaccaaaactggtcgaacaggtggc
gttaaatgcctggaaagaggagagcgacaacgcagtatgtctgcatttgcgctcctctca
gcggcatttgaacaaccgcggtgcacagcaaaaactggctgaagcgttgagcatgttaaa
aggttcaacggttgaactgactatcgttgaagatgataatcccgcggtgcgtacgccgct
ggagtggcgtcaggcgatatacgaagaaaaacttgcgcaggcgcgcgagtccattattgc
ggataataatattcagaccctgcgtcggttcttcgatgcggagctggatgaagaaagtat
ccgccccatttga
```

DnaX protein reported sequence

```
>P12B_RS02530_1 Escherichia coli P12b 512225:514157, DnaX
MSYQVLARKWRPQTFADVVGQEHVLTALANGLSLGRIHHAYLFSGTRGVGKTSIARLLAK
GLNCETGITATPCGVCDNCREIEQGRFVDLIEIDAASRTKVEDTRDLLDNVQYAPARGRF
KVYLIDEVHMLSRHSFNALLKTLEEPPEHVKFLLATTDPQKLPVTILSRCLQFHLKALDV
EQIRHQLEHILNEEHIAHEPRALQLLARAAEGSLRDALSLTDQAIASGDGQVSTQAVSAM
LGTLDDDQALSLVEAMVEANGERVMALINEAAARGIEWEALLVEMLGLLHRIAMVQLSPA
ALGNDMAAIELRMRELARTIPPTDIQLYYQTLLIGRKELPYAPDRRMGVEMTLLRALAFH
PRMPLPEPEVPRQSFAPVAPTAVMTPTQVPPQPQSAPQQAPTVPLPETTSQVLAARQQLQ
RVQGATKDKKE*TGSRYPRAAGE*RCAGKTGFGHRSRSGASGAIGAGKSASQKRSVSLEG
DHSGDAAKRSGRHAEGAEKSAGT*KNAGTGGEASGRSH*ARPVGGTGEPTFATKTGRTGG
VKCLERGERQRSMSAFALLSAAFEQPRCTAKTG*SVEHVKRFNG*TDYR*R**SRGAYAA
GVASGDIRRKTCAGARVHYCG**YSDPASVLRCGAG*RKYPPHLX
```

DnaX protein proposed sequence

```
>P12B_RS02530_1 Escherichia coli P12b 512225:514157, DnaX
MSYQVLARKWRPQTFADVVGQEHVLTALANGLSLGRIHHAYLFSGTRGVGKTSIARLLAK
GLNCETGITATPCGVCDNCREIEQGRFVDLIEIDAASRTKVEDTRDLLDNVQYAPARGRF
KVYLIDEVHMLSRHSFNALLKTLEEPPEHVKFLLATTDPQKLPVTILSRCLQFHLKALDV
EQIRHQLEHILNEEHIAHEPRALQLLARAAEGSLRDALSLTDQAIASGDGQVSTQAVSAM
LGTLDDDQALSLVEAMVEANGERVMALINEAAARGIEWEALLVEMLGLLHRIAMVQLSPA
ALGNDMAAIELRMRELARTIPPTDIQLYYQTLLIGRKELPYAPDRRMGVEMTLLRALAFH
PRMPLPEPEVPRQSFAPVAPTAVMTPTQVPPQPQSAPQQAPTVPLPETTSQVLAARQQLQ
RVQGATKAKKSEPAAATRARPVNNAALERLASVTDRVQARPVPSALEKAPAKKEAYRWKA
TTPVMQQKEVVATPKALKKALEHEKTPELAAKLAAEAIERDPWAAQVSQLSLPKLVEQVA
LNAWKEESDNAVCLHLRSSQRHLNNRGAQQKLAEALSMLKGSTVELTIVEDDNPAVRTPL
EWRQAIYEEKLAQARESIIADNNIQTLRRFFDAELDEESIRPI*
```

Deleting the nucleotide A at position 1283, we obtain this protein sequence which is 100% identical to *E. coli* K-12 strain DnaX sequence.

```
>P12B_RS07525 Escherichia coli P12b 1475870:1476414, infC
attaaaggcggaaaacgagttcaaacggcgcgccctaaccgtatcaatggcgaaattcgc
gcccaggaagttcgcttaacaggtctggaaggcgagcagcttggtattgtgagtctgaga
gaagctctggagaaagcagaagaagccggagtagacttagtcgagatcagccctaacgcc
gagccgccggtttgtcgtataatggattacggcaaattcctctatgaaaagagcaagtct
tctaaggaacagaaagaaaaagacaaaaagttatccaggttaaggaaattaaattccgtc
ctggtacagatgaaggcgactatcaggtaaaactccgcagcctgattcgctttctcgaag
agggtgataaagccaaaatcacgctgcgtttccgcggtcgtgagatggcgcaccagcaaa
tcggtatggaagtgcttaatcgcgtgaaagacgatttgcaagaactggcagtggtcgaat
ccttcccaacgaagatcgaaggccgccagatgatcatggtgctcgctcctaagaagaaac
agtaa
```

InfC protein reported sequence

```
>P12B_RS07525_1 Escherichia coli P12b 1475870:1476414, InfC
IKGGKRVQTARPNRINGEIRAQEVRLTGLEGEQLGIVSLREALEKAEEAGVDLVEISPNA
EPPVCRIMDYGKFLYEKSKSSKEQKEKDKKLSRLRKLNSVLVQMKATIR*NSAA*FAFSK
RVIKPKSRCVSAVVRWRTSKSVWKCLIA*KTICKNWQWSNPSQRRSKAAR*SWCSLLRRN
SX
```

InfC protein proposed sequence

```
>P12B_RS07525_1 Escherichia coli P12b 1475870:1476414, InfC
IKGGKRVQTARPNRINGEIRAQEVRLTGLEGEQLGIVSLREALEKAEEAGVDLVEISPNA
EPPVCRIMDYGKFLYEKSKSSKEQKKKQKVIQVKEIKFRPGTDEGDYQVKLRSLIRFLEE
GDKAKITLRFRGREMAHQQIGMEVLNRVKDDLQELAVVESFPTKIEGRQMIMVLAPKKKQ
*
```

Deleting the nucleotides A at position 255 and at position 263, obtain this prote sequence which i 100% identical t *E. coli* K12 stra InfC sequence.

```
>P12B_RS18895 Escherichia coli P12b 3713289:3713829, rplE
atggcgaaactgcatgattactacaaagacgaagtagttaaaaaactcatgactgagttt
aactacaattctgtcatgcaagtccctcgggtcgagaagatcaccctgaacatgggtgtt
ggtgaagcgatcgctgacaaaaaactagctggataacgcagcagcagacctggcagcaat
ctccggtcaaaaaccgctgatcaccaaagcacgcaaatctgttgcaggcttcaaaatccg
tcagggctatccgatcggctgtaaagtaactctgcgtggcgaacgcatgtgggagttctt
tgagcgcctgatcactattgctgtacctcgtatccgtgacttccgtggcctgtccgctaa
gtctttcgacggtcgtggtaactacagcatgggtgtccgtgagcagatcatcttcccaga
aatcgactacgataaagtcgaccgcgttcgtggtttggatattaccattaccactactgc
gaaatctgacgaagaaggccgcgctctgctggctgcctttgacttcccgttccgcaagta
a
```

RplE protein reported sequence

```
>P12B_RS18895_1 Escherichia coli P12b 3713289:3713829, RplE
MAKLHDYYKDEVVKKLMTEFNYNSVMQVPRVEKITLNMGVGEAIADKKLAG*RSSRPGSN
LRSKTADHQSTQICCRLQNPSGLSDRL*SNSAWRTHVGVL*APDHYCCTSYP*LPWPVR*
VFRRSW*LQHGCP*ADHLPRNRLR*SRPRSWFGYYHYHYCEI*RRRPRSAGCL*LPVPQV
X
```

RplE protein proposed sequence

```
>P12B_RS18895_1 Escherichia coli P12b 3713289:3713829, RplE
MAKLHDYYKDEVVKKLMTEFNYNSVMQVPRVEKITLNMGVGEAIADKKLLDNAAADLAAI
SGQKPLITKARKSVAGFKIRQGYPIGCKVTLRGERMWEFFERLITIAVPRIRDFRGLSAK
SFDGRGNYSMGVREQIIFPEIDYDKVDRVRGLDITITTTAKSDEEGRALLAAFDFPFRK*
```

Deleting the nucleotide A at position 147, we obtain this protein sequence which is 100% identical to *E. coli* K12 strain RplE sequence.

```
>P12B_RS04910 Escherichia coli P12b 996422:1000883, mukB
atgattgaacgcggtaaatttcgctcactgacgctgattaactggaacggctttttgcc
cgaacttttgaccttgacgagctggtcacgacgctttctggcggtaacggggcgggtaaa
tccaccaccatggcggcgttcgttacggcgctgatccccgacctgaccctgctgcatttc
cgtaacactacggaagccggggccaccagcggttcgcgcgataaaggtctgcacggtaag
ctgaaagcgggtgtctgttattcgatgctcgacaccattaactcgcgccaccagcgcgtg
gtggtcggtgtgcgtctgcaacaggttgccggacgcgatcgtaaagtggatatcaagccg
tttgccattcagggactgccgatgtcggtgcagccgacacagctggtgaccgaaaccctg
aacgaacgccaggcgcgcgtgctgccgcttaacgagctgaaagacaagctcgaggcgatg
gaaggcgtgcagtttaaacagttcaactccattactgattaccactcgctgatgttcgat
ctgggcatcatcgcgcgtcgtctgcgctccgcatctgaccgtagcaaattctatcgtctg
atcgaagcttcgctgtatggcgggatctccagtgccattacccgttctctgcgcgactac
ctgttgccagaaaacagcggcgtgcgtaaagcgttccaggacatggaagcggcgctgcgt
gaaaaccgtatgacgctggaagcgattcgtgtcacccagtcggaccgcgacctgtttaag
catctgatcagcgaagccaccaactacgtggcggcggactacatgcgtcacgccaacgag
cgccgtgtccatctcgacaaagccctggagtttcgtcgcgagctacatacttcgcgtcag
caactggcggctgagcagtacaaacacgtcgatatggcgcgtgagctggcagagcacaac
ggtgccgaaggcgatctggaagcggattatcaggcggccagtgatcacctgaacctggtg
caaaccgcactgcgtcagcaggagaagatcgaacgctacgaagcggatctcgatgagctg
cagatccgtctggaagagcaaaatgaagtggtggcagaagccatcgaacgccaggaagag
aatgaggctcgtgcggaagctgccgaactggaagtggacgagctgaaaagccagcttgct
gactaccagcaggcgctggacgtccagcaaacgcgcgcgatccagtataaccaggcgatt
gctgcgcttaatcgtgccaaagaactgtgccatctgccggacttaaccgccgactgcgcc
gccgaatggctggaaaccttccaggcgaaagagctggaagcgactgaaaaaatgctctct
cttgagcagaaaatgagcatggcgcaaaccgcgcacagccagtttgagcaggcttatcag
ctggtggtggcaatcaacggcccactggcgcgtaacgaggcgtgggatgtcgctcgcgaa
ctattgcgcgaaggggtcgatcagcgtcacctggcagagcaggttcagccgctgcggatg
cgattaagcgaactggaacagcgtctacgcgagcagcaagaagctgagcgtctgctggca
gatttctgcaaacgtcagggcaagaattttgatatcgacgaactggaagccctgcatcag
gaactggaagcacgcattgcctctctttccgatagcgtgtctaacgcccgtgaagagcgc
atggcactgcgccaggagcaggaacagctgcagtctcgcattcagagtttgatgcagcgt
gcgccggtttggctggcagcgcaaaacagtctcaaccagttgagcgaacagtgcggcgaa
gagtttacctccagccaggacgtcaccgaatatctgcaacagttgctggagcgtgagcga
gaggcgattgttgaacgcgatgaagtgggcgcgcgcaaaaacgccgtcgatgaagagatc
gaacgtttaagccagcctggcggctctgaagatcagcgtctgaacgcgctggcggagcgt
tttggtggtgtgctgctgtcagaaatttatgacgacgttagcctggaagatgcgccgtac
ttctcagcgctgtatggcccgtcacgccacgccatcgtggtgccagatctgtcacaggta
actgaacacctggaaggcttgaccgattgcccggaagatctctatctgatcgaaggagat
ccgcagtcattcgatgacagcgtgttcagcgttgatgagctggaaaaagcggtagtggtg
aaaatcgccgatcgtcagtggcgttattcacgtttcccggaagtgccgctgtttggtcgt
gctgcgcgtgaaagccgtattgaaagcctccatgccgagcgtgaagtgctttccgaacgc
ttcgccacgctctcctttgatgtacagaaaactcagcgtctgcatcaggcgttcagccgc
tttatcggcagtcatctggcggttgcgtttgagtctgacccggaagcagaaatccgtcaa
ctgaacagccgtcgcgtcgaactggagcgggcgttaagtaatcatgaaaatgataaccag
cagcagcgtattcagtttgagcaggcgaaagagggcgttacggcgctgaaccgcattctg
ccgcgtctcaacctgttggctgatgacagcctggcggatcgcgtcgatgaaatccgcgaa
cgtctggatgaagcccaggaagccgcgcgttttgttcagcagtttggcaatcaactggcg
aaactggaaccgattgtttcggtattgcagagcgacccggaacagttcgaacagttaaaa
gaagattacgcgtactctcagcagatgcagcgcgatgcccgtcagcaggcgtttgccctg
acggaagtggtgcagcgtcgtgcgcactttagctattctgactcggcagaaatgcttagc
ggtaacagcgatctcaacgaaaaactgcgtgaacgtctggaacaggcggaagcggagcgt
acccgcgctcgcgaagcgttgcgcggtcacgcagcgcagttgagtcagtacaaccaggtg
ctggcttcgctgaaaagttcttacgacaccaaaaaagagctactcaacgatctgcaacg
tgaattgcaggatatcggcgtgcgtgctgatagcggggcagaagagcgggcgcgtattcg
ccgtgacgagctgcatgcgcaactgagcaataaccgttcacgccgcaatcaactggaaaa
agcgcttaccttctgcgaagcggagatggacaacctgacccgcaaactgcgcaagctgga
gcgggattactttgagatgcgcgagcaggtagtgaccgccaaagcgggctggtgtgcggt
gatgcgcatggtgaaagataacggcgttgagcgccgcttacaccgtcgtgagctggctta
tctctccgctgatgatttgcgttccatgtcggataaggcgttaggtgcgctgcgtctggc
ggtggcggataacgaacatctgcgcgacgtgctgcgcatgtcggaagatccgaaacgtcc
```

```
ggagcgtaaaattcagttcttcgtggcggtttatcagcatctgcgtgaacgtattcgtca
ggatattattcgtaccgatgatccggtggaagctatcgaacagatggagattgaacttag
ccgtctgaccgaagaattaacctcccgtgaacagaaactggcgatcagttcccgcagcgt
ggcgaacatcattcgcaaaaccattcagcgcgagcagaaccgtatccgtatgctcaacca
ggggttgcagaacgtatcgtttggtcaggtgaacagcgtgcgtctcaacgtgaacgtgcg
tgaaacgcacgccatgctactggatgtgctctctgaacagcacgagcagcatcaggatct
gtttaacagcaaccgtttgaccttctcggaagcgctggcgaaactgtatcaacgtcttaa
cccgcagattgatatggggcagcgcacgccgcagaccatcggtgaagaactgctggatta
ccgcaactatctggaaatggaagttgaggttaaccgtggttccgatggctggctgcgcgc
agagtctggtgcattgtcgaccggtgaggcgattggtaccggtatgtcgattctggtgat
ggtggtacaaagctgggaagatgaatctcgccgcctgcgcggtaaagatatctctccttg
ccgcctgctgttcctcgatgaagcagcgcgactggatgctcgttctatcgccacgctgtt
tgaattgtgtgagcgtttgcaaatgcaactcatcatcgcagcgccggaaaatatcagccc
ggagaaaggcaccacctataaactggtgcgtaaagtcttccagaataccgaacacgttca
tgtcgtcggcctgcgaggatttgcgccgcaactccctgaaacgcttccaggaactgacga
agcgccttctcaggcgagttaa
```

MukB protein reported sequence

```
>P12B_RS04910_1 Escherichia coli P12b 996422:1000883, MukB
MIERGKFRSLTLINWNGFFARTFDLDELVTTLSGGNGAGKSTTMAAFVTALIPDLTLLHF
RNTTEAGATSGSRDKGLHGKLKAGVCYSMLDTINSRHQRVVVGVRLQQVAGRDRKVDIKP
FAIQGLPMSVQPTQLVTETLNERQARVLPLNELKDKLEAMEGVQFKQFNSITDYHSLMFD
LGIIARRLRSASDRSKFYRLIEASLYGGISSAITRSLRDYLLPENSGVRKAFQDMEAALR
ENRMTLEAIRVTQSDRDLFKHLISEATNYVAADYMRHANERRVHLDKALEFRRELHTSRQ
QLAAEQYKHVDMARELAEHNGAEGDLEADYQAASDHLNLVQTALRQQEKIERYEADLDEL
QIRLEEQNEVVAEAIERQEENEARAEAAELEVDELKSQLADYQQALDVQQTRAIQYNQAI
AALNRAKELCHLPDLTADCAAEWLETFQAKELEATEKMLSLEQKMSMAQTAHSQFEQAYQ
LVVAINGPLARNEAWDVARELLREGVDQRHLAEQVQPLRMRLSELEQRLREQQEAERLLA
DFCKRQGKNFDIDELEALHQELEARIASLSDSVSNAREERMALRQEQEQLQSRIQSLMQR
APVWLAAQNSLNQLSEQCGEEFTSSQDVTEYLQQLLEREREAIVERDEVGARKNAVDEEI
ERLSQPGGSEDQRLNALAERFGGVLLSEIYDDVSLEDAPYFSALYGPSRHAIVVPDLSQV
TEHLEGLTDCPEDLYLIEGDPQSFDDSVFSVDELEKAVVVKIADRQWRYSRFPEVPLFGR
AARESRIESLHAEREVLSERFATLSFDVQKTQRLHQAFSRFIGSHLAVAFESDPEAEIRQ
LNSRRVELERALSNHENDNQQQRIQFEQAKEGVTALNRILPRLNLLADDSLADRVDEIRE
RLDEAQEAARFVQQFGNQLAKLEPIVSVLQSDPEQFEQLKEDYAYSQQMQRDARQQAFAL
TEVVQRRAHFSYSDSAEMLSGNSDLNEKLRERLEQAEAERTRAREALRGHAAQLSQYNQV
LGFAEKFLRHQKRATQRSAT*IAGYRRAC**RGRRAGAYSP*RAACATEQ*PFTPQSTGK
SAYLLRSGDGQPDPQTAQAGAGLL*DARAGSDRQSGLVCGDAHGER*RR*APLTPS*AGL
SLR**FAFHVG*GVRCAASGGGG*RTSARRAAHVGRSETSGA*NSVLRGGLSASA*TYSS
GYYSYR*SGGSYRTDGD*T*PSDRRINLP*TETGDQFPQRGEHHSQNHSARAEPYPYAQP
GVAERIVWSGEQRASQRERA*NARHATGCAL*TARAASGSV*QQPFDLLGSAGETVSTS*
PAD*YGAAHAADHR*RTAGLPQLSGNGS*G*PWFRWLAARRVWCIVDR*GDWYRYVDSGD
GGTKLGR*ISPPAR*RYLSLPPAVPR*SSATGCSFYRHAV*IV*AFANATHHRSAGKYQP
GERHHL*TGA*SLPEYRTRSCRRPARICAATP*NASRN*RSAFSGELX
```

MukB
protein
proposed
sequence

>P12B_RS04910_1 Escherichia coli P12b 996422:1000883, MukB
MIERGKFRSLTLINWNGFFARTFDLDELVTTLSGGNGAGKSTTMAAFVTALIPDLTLLHF
RNTTEAGATSGSRDKGLHGKLKAGVCYSMLDTINSRHQRVVVGVRLQQVAGRDRKVDIKP
FAIQGLPMSVQPTQLVTETLNERQARVLPLNELKDKLEAMEGVQFKQFNSITDYHSLMFD
LGIIARRLRSASDRSKFYRLIEASLYGGISSAITRSLRDYLLPENSGVRKAFQDMEAALR
ENRMTLEAIRVTQSDRDLFKHLISEATNYVAADYMRHANERRVHLDKALEFRRELHTSRQ
QLAAEQYKHVDMARELAEHNGAEGDLEADYQAASDHLNLVQTALRQQEKIERYEADLDEL
QIRLEEQNEVVAEAIERQEENEARAEAAELEVDELKSQLADYQQALDVQQTRAIQYNQAI
AALNRAKELCHLPDLTADCAAEWLETFQAKELEATEKMLSLEQKMSMAQTAHSQFEQAYQ
LVVAINGPLARNEAWDVARELLREGVDQRHLAEQVQPLRMRLSELEQRLREQQEAERLLA
DFCKRQGKNFDIDELEALHQELEARIASLSDSVSNAREERMALRQEQEQLQSRIQSLMQR
APVWLAAQNSLNQLSEQCGEEFTSSQDVTEYLQQLLEREREAIVERDEVGARKNAVDEEI
ERLSQPGGSEDQRLNALAERFGGVLLSEIYDDVSLEDAPYFSALYGPSRHAIVVPDLSQV
TEHLEGLTDCPEDLYLIEGDPQSFDDSVFSVDELEKAVVVKIADRQWRYSRFPEVPLFGR
AARESRIESLHAEREVLSERFATLSFDVQKTQRLHQAFSRFIGSHLAVAFESDPEAEIRQ
LNSRRVELERALSNHENDNQQQRIQFEQAKEGVTALNRILPRLNLLADDSLADRVDEIRE
RLDEAQEAARFVQQFGNQLAKLEPIVSVLQSDPEQFEQLKEDYAYSQQMQRDARQQAFAL
TEVVQRRAHFSYSDSAEMLSGNSDLNEKLRERLEQAEAERTRAREALRGHAAQLSQYNQV
LASLKSSYDTKKELLNDLQRELQDIGVRADSGAEERARIRRDELHAQLSNNRSRRNQLEK
ALTFCEAEMDNLTRKLRKLERDYFEMREQVVTAKAGWCAVMRMVKDNGVERRLHRRELAY
LSADDLRSMSDKALGALRLAVADNEHLRDVLRMSEDPKRPERKIQFFVAVYQHLRERIRQ
DIIRTDDPVEAIEQMEIELSRLTEELTSREQKLAISSRSVANIIRKTIQREQNRIRMLNQ
GLQNVSFGQVNSVRLNVNVRETHAMLLDVLSEQHEQHQDLFNSNRLTFSEALAKLYQRLN
PQIDMGQRTPQTIGEELLDYRNYLEMEVEVNRGSDGWLRAESGALSTGEAIGTGMSILVM
VVQSWEDESRRLRGKDISPCRLLFLDEAARLDARSIATLFELCERLQMQLIIAAPENISP
EKGTTYKLVRKVFQNTEHVHVVGLRGFAPQLPETLPGTDEAPSQAS*

Deleting the nucleotide G at position 3065, we obtain this protein sequence which is 99.93% identical to *E. coli* K12 strain MukB sequence.

```
>P12B_RS04750 Escherichia coli P12b 959522:960815, serS
atgctcgatcccaatctgctgcgtaatgagccagacgcagtcgctgaaaaactggcacgc
cggggctttaagctggatgtagataagctgggcgctcttgaagagcgtcgtaaagtattg
caggtcaaaacggaaaacctgcaagcggagcgtaactcccgatcgaaatccattggccag
gcgaaagcgcgcggggaagatatcgagcctttacgtctggaagtgaacaaactgggcgaa
gagctggatgcagcaaaagccgagctggatgctttacaggctgaaattcgcgatatcgcg
ctgaccatccctaacctgcctgcagatgaagtgccggtaggtaaagacgaaaatgacaac
gttgaagtcagccgctggggtaccccgcgtgagtttgactttgaagttcgtgaccacgtg
acgctgggtgaaatgcactctggcctcgactttgcagctgcagttaagctgactggttcc
cgctttgtggtaatgaaagggcagattgctcgcatgcaccgcgcactgtcgcagtttatg
ctggatctgcataccgaacagcatggctacagtgagaactatgttccgtacctggttaac
caggaccacgctgtacggtacgggtcaactgccgaaatttgctggcgatctgttccatac
tcgtccgctggaagaagaagcagacaccagtaactatgcgctgatcccaacggcagaagt
tccgctgactaacctggtgcgcggtgaaatcatcgatgaagatgatctgccaattaagat
gaccgcccacaccccatgcttccgttctgaagccggttcatatggtcgtgacacccgtgg
tctgatccgtatgcaccagttcgacaaagttgaaatggtgcagatcgtgcgcccagaaga
ctcaatggcggcgctggaagagatgactggtcatgcagaaaaagtcctgcagttgctggg
cctgccgtaccgtaaaatcatcctttgcactggcgacatgggctttggcgcttgcaaaac
ttacgacctggaagtatggatcccggcacagaacacctaccgtgagatctcttcctgctc
caacgtttgggatttccaggcacgtcgtatgcaggcacgttgccgcagcaagtcggacaa
gaaaacccgtctggttcataccctgaacggttctggtctggctgttggtcgtacgctggt
tgcagtaatggaaaactatcagcaggctgatggtcgtattgaagtaccagaagttctgcg
tccgtatatgaacggactggaatatattggctaa
```

SerS protein reported sequence

```
>P12B_RS04750_1 Escherichia coli P12b 959522:960815, SerS
MLDPNLLRNEPDAVAEKLARRGFKLDVDKLGALEERRKVLQVKTENLQAERNSRSKSIGQ
AKARGEDIEPLRLEVNKLGEELDAAKAELDALQAEIRDIALTIPNLPADEVPVGKDENDN
VEVSRWGTPREFDFEVRDHVTLGEMHSGLDFAAAVKLTGSRFVVMKGQIARMHRALSQFM
LDLHTEQHGYSENYVPYLVNQDHAVRYGSTAEICWRSVPYSSAGRRSRHQ*LCADPNGRS
SAD*PGAR*NHR*R*SAN*DDRPHPMLPF*SRFIWS*HPWSDPYAPVRQS*NGADRAPRR
LNGGAGRDDWSCRKSPAVAGPAVP*NHPLHWRHGLWRLQNLRPGSMDPGTEHLP*DLFLL
QRLGFPGTSYAGTLPQQVGQENPSGSYPERFWSGCWSYAGCSNGKLSAG*WSY*STRSSA
SVYERTGIYWLX
```

SerS protein proposed sequence

```
>P12B_RS04750_1 Escherichia coli P12b 959522:960815, SerS
MLDPNLLRNEPDAVAEKLARRGFKLDVDKLGALEERRKVLQVKTENLQAERNSRSKSIGQ
AKARGEDIEPLRLEVNKLGEELDAAKAELDALQAEIRDIALTIPNLPADEVPVGKDENDN
VEVSRWGTPREFDFEVRDHVTLGEMHSGLDFAAAVKLTGSRFVVMKGQIARMHRALSQFM
LDLHTEQHGYSENYVPYLVNQDTLYGTGQLPKFAGDLFHTRPLEEEADTSNYALIPTAEV
PLTNLVRGEIIDEDDLPIKMTAHTPCFRSEAGSYGRDTRGLIRMHQFDKVEMVQIVRPED
SMAALEEMTGHAEKVLQLLGLPYRKIILCTGDMGFGACKTYDLEVWIPAQNTYREISSCS
NVWDFQARRMQARCRSKSDKKTRLVHTLNGSGLAVGRTLVAVMENYQQADGRIEVPEVLR
PYMNGLEYIG*
```

Deleting the nucleotide C at position 607, we[illegible] obtain this prot[illegible] sequence which [illegible] 100% identical t[illegible] *E. coli* K12 stra[illegible] SerS sequence.

```
> Escherichia coli UM146 4871474:4874178, secA
atgctaatcaaattattaactaaagttttcggtagtcgtaacgatcgcaccctgcgccgg
atgcgcaaagtggtcaacatcatcaatgccatggaaccggagatggaaaaactctccgac
gaagaactgaaagggaaaaccgcagagtttcgtgcgcgtctggaaaaaggcgaagtgctg
gaaaatctgatcccggaagctttcgccgtggtgcgtgaggcaagtaagcgcgtctttggc
atgcgtcacttcgacgttcagttactcggcggtatggttcttaacgaacgctgcatcgcc
gaaatgcgtaccggtgaaggtaaaaccctgaccgcaacgctgcctgcttacctgaacgca
ctaaccggtaaaggcgtacacgtagttaccgtcaacgactacctggcgcaacgtgacgcc
gaaaacaaccgtccgctgtttgaattccttggcctgactgtcggtatcaacctgccgggc
atgccagcaccggcaaagcgtgaagcctacgctgctgacatcacttacggtacgaacaac
gaatacggctttgactacctgcgtgacaacatggcgttcagtcctgaagaacgtgtacag
cgtaaactgcactatgcgctggtggacgaagtggactccatcctgatcgatgaagcgcgt
acaccgctgatcatttccggcccggcagaagacagctcggaaatgtataaacgcgtgaat
aaaattattccgcacctgatccgtcaggaaaaagaagactccgaaaccttccagggcgaa
ggccacttctcggtggatgaaaaatctcgccaggtgaacctgaccgaacgtggtctggtc
ctgattgaagaactgctggttaaagaaggcatcatggatgaaggagagtctctgtactct
ccggccaacatcatgctgatgcaccacgtaacggcggcgctgcgcgctcatgcgctgttt
acccgtgacgtcgactacatcgttaaagatggtgaagttatcatcgttgacgaacacacc
ggtcgtaccatgcagggccgtcgctggtccgatggtctgcaccaggctgtggaagcgaaa
gaaggtgtgcagatccagaacgaaaaccaaacgctggcttcgatcaccttccagaactac
ttccgtctgtatgaaaaactggcggggatgactggtactgctgataccgaagctttcgaa
ttcagctccatctataagctggatactgtcgttgttccgaccaaccgcccaatgattcgt
aaagatctgccggacctggtctacatgactgaagcggaaaaaattcaggcgatcattgaa
gatatcaaagaacgtactgcgaaaggccagccggtgctggtgggtacaatctccatcgaa
aaatcggagctggtgtcaaatgaactgaccaaagccggtattaagcacaacgtcctgaac
gccaaattccatgccaacgaagcggcgattgttgctcaggcaggttacccggctgcggtg
actatcgcgaccaacatggcgggtcgtggtaccgatattgtgctcggtggtagctggcag
gcagaagttgccgcgctggaaaatccgactgcagagcaaattgaaaaattaaagccgact
ggcaggtacgtcacgatgcggtactggcagcaggtggcctgcatatcatcggtactgaac
gtcacgaatcccgtcgtatcgataaccagctgcgcggtcgttctggtcgtcaggggggatg
ctggttcttcccgtttctacctgtcgatggaagatgcgctgatgcgtattttttgcttccg
accgagtatccggcatgatgcgtaaactgggtatgaagccaggcgaagccattgagcacc
cgtgggtgaccaaagcgattgccaacgcccagcgtaaagttgaaagccgtaacttcgaca
ttcgtaagcaactgctggaatatgatgacgtggctaacgatcagcgtcgcgccatttact
cccagcgtaacgaactgctggatgtcagcgatgtgagcgaaaccatcaacagcattcgtg
aagatgtgttcaaagcgaccattgatgcctacattccgccacagtcgctggaagaaatgt
gggatattccggggctgcaggaacgtctgaagaacgatttcgacctcgatttaccaattg
ccgagtggctggataaagaaccagaactgcatgaagagacgctgcgtgagcgcattctgg
cgcagtccatcgaagtgtatcagcgtaaagaagaagtggttggtgctgagatgatgcgtc
acttcgagaaaggcgtcatgctgcaaactctcgactctctgtggaaagagcacctggcag
cgatggactatctgcgtcagggtatccacctgcgtggctatgcacagaaagatccgaagc
aggaatacaaacgtgaatcgttctccatgtttgcagcgatgctggagtcgttgaaatatg
aagttatcagtacgctgagcaaagttcaggtacgtatgcctgaagaggttgaggagctgg
aacaacagcgtcgtatggaagccgagcgtttagcgcaaatgcagcagcttagctatcagg
atgacgactctgcagccgcagctgcactggcggcacaaaccggtgaacgcaaagtaggac
gtaacgatccttgcccgtgtggttctggtaaaaaatacaagcagtgccatggccgcctgc
aataa
```

SecA protein reported sequence

>Escherichia coli UM146 4871474:4874178, SecA
MLIKLLTKVFGSRNDRTLRRMRKVVNIINAMEPEMEKLSDEELKGKTAEFRARLEKGEVL
ENLIPEAFAVVREASKRVFGMRHFDVQLLGGMVLNERCIAEMRTGEGKTLTATLPAYLNA
LTGKGVHVVTVNDYLAQRDAENNRPLFEFLGLTVGINLPGMPAPAKREAYAADITYGTNN
EYGFDYLRDNMAFSPEERVQRKLHYALVDEVDSILIDEARTPLIISGPAEDSSEMYKRVN
KIIPHLIRQEKEDSETFQGEGHFSVDEKSRQVNLTERGLVLIEELLVKEGIMDEGESLYS
PANIMLMHHVTAALRAHALFTRDVDYIVKDGEVIIVDEHTGRTMQGRRWSDGLHQAVEAK
EGVQIQNENQTLASITFQNYFRLYEKLAGMTGTADTEAFEFSSIYKLDTVVVPTNRPMIR
KDLPDLVYMTEAEKIQAIIEDIKERTAKGQPVLVGTISIEKSELVSNELTKAGIKHNVLN
AKFHANEAAIVAQAGYPAAVTIATNMAGRGTDIVLGGSWQAEVAALENPTAEQIEKLKPT
GRYVTMRYWQQVACISSVLNVTNPVVSITSCAVVLVVRGMLVLPVSTCRWKMR*CVFLLP
TEYPA*CVNWV*SQAKPLSTRG*PKRLPTPSVKLKAVTSTFVSNCWNMMTWLTISVAPFT
PSVTNCWMSAM*AKPSTAFVKMCSKRPLMPTFRHSRWKKCGIFRGCRNV*RTISTSIYQL
PSGWIKNQNCMKRRCVSAFWRSPSKCISVKKKWLVLR*CVTSRKASCCKLSTLCGKSTWQ
RWTICVRVSTCVAMHRKIRSRNTNVNRSPCLQRCWSR*NMKLSVR*AKFRYVCLKRLRSW
NNSVVWKPSV*RKCSSLAIRMTTLQPQLHWRHKPVNAK*DVTILARVVLVKNTSSAMAAC
NX

SecA protein proposed sequence

>Escherichia coli UM146 4871474:4874178, SecA
MLIKLLTKVFGSRNDRTLRRMRKVVNIINAMEPEMEKLSDEELKGKTAEFRARLEKGEVL
ENLIPEAFAVVREASKRVFGMRHFDVQLLGGMVLNERCIAEMRTGEGKTLTATLPAYLNA
LTGKGVHVVTVNDYLAQRDAENNRPLFEFLGLTVGINLPGMPAPAKREAYAADITYGTNN
EYGFDYLRDNMAFSPEERVQRKLHYALVDEVDSILIDEARTPLIISGPAEDSSEMYKRVN
KIIPHLIRQEKEDSETFQGEGHFSVDEKSRQVNLTERGLVLIEELLVKEGIMDEGESLYS
PANIMLMHHVTAALRAHALFTRDVDYIVKDGEVIIVDEHTGRTMQGRRWSDGLHQAVEAK
EGVQIQNENQTLASITFQNYFRLYEKLAGMTGTADTEAFEFSSIYKLDTVVVPTNRPMIR
KDLPDLVYMTEAEKIQAIIEDIKERTAKGQPVLVGTISIEKSELVSNELTKAGIKHNVLN
AKFHANEAAIVAQAGYPAAVTIATNMAGRGTDIVLGGSWQAEVAALENPTAEQIEKIKAD
WQVRHDAVLAAGGLHIIGTERHESRRIDNQLRGRSGRQGDAGSSRFYLSMEDALMRIFAS
DRVSGMMRKLGMKPGEAIEHPWVTKAIANAQRKVESRNFDIRKQLLEYDDVANDQRRAIY
SQRNELLDVSDVSETINSIREDVFKATIDAYIPPQSLEEMWDIPGLQERLKNDFDLDLPI
AEWLDKEPELHEETLRERILAQSIEVYQRKEEVVGAEMMRHFEKGVMLQTLDSLWKEHLA
AMDYLRQGIHLRGYAQKDPKQEYKRESFSMFAAMLESLKYEVISTLSKVQVRMPEEVEEL
EQQRRMEAERLAQMQQLSYQDDDSAAAAALAAQTGERKVGRNDPCPCGSGKKYKQCHGRL
Q*

Adding the nucleotide A at position 1609, we obtain this protei sequence which is 99.78% identical t *E. coli* K12 SecA sequence.

>APECO1_RS14765 Escherichia coli APEC01 2903796:2904565, trmD
atgtggattggcataattagcctgtttcctgaaatgttccgcgcaattaccgattacggg
gtaactggccgggcagttaaaaatggcctgctgagcatccagagctggagtcctcgcgac
ttcacgcatgaccggcaccgtaccgtggacgatcgtccttacggcggcggaccggggatg
ttaatgatggtgcaacccttgcgggacgccattcatgcagcaaaagccgcggcgggtgaa
ggcgcaaaggtgatttatctgtcaccacagggacgcaagcttgatcaagcgggcgtcagc
gaactggcaacgaatcaaaaattgattctggtgtgtggtcgctacgaaaggtatagatga
gcgcgtgatccaaaccgaaaattgacgaagaatggtcaatcggcgattacgttctcagtg
gtggtgagttaccagcaatgacgctgattgactccgtttcccggtttattccgggagtac
tgggacatgaagcctcggcaacggaagattcctttgctgaaggattgctggattgcccgc
actatacgcggcctgaggtgttagaagggatggaagttccgccagtgttactgtcgggta
accatgccgagatacgtcgctggcgtttgaaacagtcgctgggccgtacctggcttagaa
gacctgaacttctggaaaacctggctctgactgaagagcaagcaaggttgctggcggagt
tcaaaacggaacacgcacaacagcaacataaacatgatggatggcgtaa

TrmD protein reported sequence

>APECO1_RS14765_1 Escherichia coli APEC01 2903796:2904565, TrmD
MWIGIISLFPEMFRAITDYGVTGRAVKNGLLSIQSWSPRDFTHDRHRTVDDRPYGGGPGM
LMMVQPLRDAIHAAKAAAGEGAKVIYLSPQGRKLDQAGVSELATNQKLILVCGRYERYR*
ARDPNRKLTKNGQSAITFSVVVSYQQ*R*LTPFPGLFREYWDMKPRQRKIPLLKDCWIAR
TIRGLRC*KGWKFRQCYCRVTMPRYVAGV*NSRWAVPGLEDLNFWKTWL*LKSKQGCWRS
SKRNTHNSNINMMGWRX

TrmD protein proposed sequence

>APECO1_RS14765_1 Escherichia coli APEC01 2903796:2904565, TrmD
MWIGIISLFPEMFRAITDYGVTGRAVKNGLLSIQSWSPRDFTHDRHRTVDDRPYGGGPGM
LMMVQPLRDAIHAAKAAAGEGAKVIYLSPQGRKLDQAGVSELATNQKLILVCGRYEGIDE
RVIQTEIDEEWSIGDYVLSGGELPAMTLIDSVSRFIPGVLGHEASATEDSFAEGLLDCPH
YTRPEVLEGMEVPPVLLSGNHAEIRRWRLKQSLGRTWLRRPELLENLALTEEQARLLAEF
KTEHAQQQHKHDGMA*

Deleting the nucleotides A and A at positions 349 and 381 respectively, we obtain this protein sequence which is 100% identical to *E. coli* K12 TrmD sequence.

```
>ECP_4259 Escherichia coli 536 4467082:4468353, plsB
atgtccggctggccacgaatttactacaaattactgaatttaccattaagcatcctggta
aaaagcaagtctattccggcggatcctgccccggaactggggctggatacctctcgtcca
attatgtacgttttaccgtacaactcgaaagctgacttgctgacgttgcgcgcccagtgt
ctggcacatgacctgcctgacccgttagagccgctggaaatcgacggcacgctactgccg
cgctatgtgttcattcacggcgggccgcgtgtgttcacctattacacgccgaaagaagag
tctattaagctgttccacgactatctcgatttgcaccgcagcaacccaaatctggatgtg
cagatggtgccagtatcggtgatgtttggtcgcgcgccggggcgtgaaaaaggcgaagtg
aacccgccgctgcgtatgcttaacggtgtacagaaatttttcgctgtactgtggctcggt
cgcgacagttttgtgcgtttctcgccgtcagtttcgctgcgccgtatggcggatgaacac
ggcacggataaaactatcgcgcagaaactggcgcgcgtggcgcgtatgcactttgcccgt
caacgtctggctgccgtaggaccacgtctgcctgctcgtcaggatctgtttaataagctg
ctcgcctcccgcgccattgccaaagcggtagaagatgaagcgcgcagcaaaaaaatctcc
catgaaaaagcgcagcagaacgcgattgcgctgatggaagagattgcggcgaatttctct
tacgagatgatccgcctgactgaccgcattctgggcttcacctggaaccgactttaccag
ggtatcaacgtccataacgccgagcgcgttcgccagctggcccacgacggccatgagcta
gtgtatgtgccttgccaccgcagtcacatggactacctgctgctttcttacgtgctgtat
caccaggggctggtgccgccgcatatcgccgccgggatcaacctgaactttttggccggct
gggccgattttccgccgtctgggggcgttctttattcgccgtacgtttaaaggcaataaa
ctttattccaccgttttccgtgagtatctcggcgaactgttcagccgtggttattccgtc
gagtatttcgtggaaggcggtcgttcccgtacggggcgttgctggatccgaaaaccggta
cgctgtcgatga
```

PlsB protein reported sequence

```
>c4468353-4465871_1 Escherichia coli 536, PlsB
MTFCYPCRAFALLTRGFTSFMSGWPRIYYKLLNLPLSILVKSKSIPADPAPELGLDTSRP
IMYVLPYNSKADLLTLRAQCLAHDLPDPLEPLEIDGTLLPRYVFIHGGPRVFTYYTPKEE
SIKLFHDYLDLHRSNPNLDVQMVPVSVMFGRAPGREKGEVNPPLRMLNGVQKFFAVLWLG
RDSFVRFSPSVSLRRMADEHGTDKTIAQKLARVARMHFARQRLAAVGPRLPARQDLFNKL
LASRAIAKAVEDEARSKKISHEKAQQNAIALMEEIAANFSYEMIRLTDRILGFTWNRLYQ
GINVHNAERVRQLAHDGHELVYVPCHRSHMDYLLLSYVLYHQGLVPPHIAAGINLNFWPA
GPIFRRLGAFFIRRTFKGNKLYSTVFREYLGELFSRGYSVEYFVEGGRSRTGRCWIRKPV
RCR*PFRRCCVAALVRLR*FRSISVMSTSWKWVLTPKNCAVRRKRKRACRRCCVV*ASCV
ISVRGTSTSVNQCR**PTLTSTYQTGVNLSIPSKRCVRPG*RRRSIILLPI*WYALTTQA
RQTP*TCAVLRFWRHVSAHSPASS*PSNSTATWI*CATCLTPRTLPFLQPAPASLSITRC
K*TSLKSRKTLSATSSFCRASKRC**PTIATTLHICWCCLR*WRQLSPSIATSPATY*WS
TSMCFTQC*KRNCSCAGIATSYRTLLMRWQMRCNVRG*LPCKMMSCISTRRILARYSCWP
QARAKRCNVMPSPSGC*VPTRRSTAVHWRKRAAPSRNVSPCCTVSTRRSSSTRRCSVLWC
*RCVMKGISAIAATPNRQKR*RYISCWRS*LHQTCV*RLRVRRRAKGX
```

PlsB protein proposed sequence

```
>c4468353-4465871_1 Escherichia coli 536, PlsB
MSGWPRIYYKLLNLPLSILVKSKSIPADPAPELGLDTSRPIMYVLPYNSKADLLTLRAQC
LAHDLPDPLEPLEIDGTLLPRYVFIHGGPRVFTYYTPKEESIKLFHDYLDLHRSNPNLDV
QMVPVSVMFGRAPGREKGEVNPPLRMLNGVQKFFAVLWLGRDSFVRFSPSVSLRRMADEH
GTDKTIAQKLARVARMHFARQRLAAVGPRLPARQDLFNKLLASRAIAKAVEDEARSKKIS
HEKAQQNAIALMEEIAANFSYEMIRLTDRILGFTWNRLYQGINVHNAERVRQLAHDGHEL
VYVPCHRSHMDYLLLSYVLYHQGLVPPHIAAGINLNFWPAGPIFRRLGAFFIRRTFKGNK
LYSTVFREYLGELFSRGYSVEYFVEGGRSRTGRLLDPKTGTLSMTIQAMLRGGTRPITLI
PIYIGYEHVMEVGTYAKELRGATKEKESLPQMLRGLSKLRNLGQGYVNFGEPMPLMTYLN
QHVPDWRESIDPIEAVRPAWLTPTVNNIAADLMVRINNAGAANAMNLCCTALLASRQRSL
TREQLTEQLNCYLDLMRNVPYSTDSTVPSASASELIDHALQMNKFEVEKDTIGDIIILPR
EQAVLMTYYRNNIAHMLVLPSLMAAIVTQHRHISRDVLMEHVNVLYPMLKAELFLRWDRD
ELPDVIDALANEMQRQGLITLQDDELHINPAHSRTLQLLAAGARETLQRYAITFWLLSAN
PSINRGTLEKESRTVAQRLSVLHGINAPEFFDKAVFSSLVLTLRDEGYISDSGDAEPAET
MKVYQLLAELITSDVRLTIESATQGEG*
```

Adding C and T at positions 1072 and 1180 respectively and deleting the nucleotide C at position 1068, we obtain this protein sequence which is 100% identical to *E. coli* K12 strain PlsB sequence.
